# *Piper betle* L. Modulates Senescence-Associated Genes Expression in Replicative Senescent Human Diploid Fibroblasts

**DOI:** 10.1155/2017/6894026

**Published:** 2017-05-17

**Authors:** Lina Wati Durani, Shy Cian Khor, Jen Kit Tan, Kien Hui Chua, Yasmin Anum Mohd Yusof, Suzana Makpol

**Affiliations:** ^1^Department of Biochemistry, Universiti Kebangsaan Malaysia Medical Centre, Level 17, Preclinical Building, Jalan Yaacob Latif, Bandar Tun Razak, 56000 Cheras, Kuala Lumpur, Malaysia; ^2^Department of Physiology, Universiti Kebangsaan Malaysia Medical Centre, Level 18, Preclinical Building, Jalan Yaacob Latif, Bandar Tun Razak, 56000 Cheras, Kuala Lumpur, Malaysia

## Abstract

*Piper betle *(PB) is a traditional medicine that is widely used to treat different diseases around Asian region. The leaf extracts contain various bioactive compounds, which were reported to have antidiabetic, antibacterial, anti-inflammatory, antioxidant, and anticancer effects. In this study, the effect of PB aqueous extracts on replicative senescent human diploid fibroblasts (HDFs) was investigated by determining the expressions of senescence-associated genes using quantitative PCR. Our results showed that PB extracts at 0.4 mg/ml can improve cell proliferation of young (143%), presenescent (127.3%), and senescent (157.3%) HDFs. Increased expressions of* PRDX6*,* TP53*,* CDKN2A*,* PAK2*, and* MAPK14 *were observed in senescent HDFs compared to young and/or presenescent HDFs. Treatment with PB extracts modulates the transcriptional profile changes in senescent HDFs. By contrast, expressions of* SOD1 *increased, whereas* GPX1*,* PRDX6*,* TP53*,* CDKN2A*,* PAK2*, and* MAPK14* were decreased in PB-treated senescent HDFs compared to untreated senescent HDFs. In conclusion, this study indicates the modulation of PB extracts on senescence-associated genes expression of replicative senescent HDFs. Further studies warrant determining the mechanism of PB in modulating replicative senescence of HDFs through these signaling pathways.

## 1. Introduction

In 1961, Hayflick and Moorhead discovered the finite cell growth of fibroblasts after multiple division, later called replicative senescence, which serves as model to study aging [1]. Replicative senescence is an irreversible growth arrest due to limited cell expansion number which was observed in human diploid fibroblasts (HDFs) and other cell types including astrocytes [2] and smooth muscle cells [3]. Regardless of the cell types, replicative senescence affects normal biological system and is represented by various classical features. For instance, cell becomes enlarged and flattened [2, 4], with increased activity of senescence-associated beta-galactosidase (SA-*β*-Gal) [5], increased damaged DNA, and shortening of telomere length and ultimately cells are growth-arrested [6].

Free radical theory of aging postulated the involvement of oxidative stress in aging development [7]. Increased oxidative stress is attributed by the endogenous and exogenous free radicals formation due to normal metabolism and exposure to environmental oxidants [8]. Therefore, a balance between production of free radicals and cellular antioxidants defence is required in preventing oxidative stress. Antioxidants can either naturally present in our body, in the form of enzymes such as superoxide dismutases (SODs), catalase (CAT), and glutathione peroxidase (GPX), or consumed from the diet such as vitamin E, vitamin C, and carotenoids [9]. Elevated free radicals generation and ineffective antioxidant defence have been observed with increasing age. Excessive free radical impairs the cellular redox status by either causing irreversible damage to DNA, protein, and lipid or interfering with the regulation of redox signaling at transcriptional or translational levels [10]. Thus, antioxidant supplementation becomes popular, with the intention to improve the redox balance, achieve the desired longevity, and increase health span. However, does this antioxidant supplementation work as expected as antiaging agents and what is the mechanism involved?

More recently, herbal plants have gained worldwide popularity as antioxidant supplements to combat oxidative damage and act as antiaging agent [11, 12]. Betel vine (*Piper betle *Linn.; PB) is a member of Piperaceae family available in South and Southeast Asia including Malaysia throughout the year. This perennial creeper plant has glossy heart shaped and yellowish green leaves with a strong pungent aromatic flavor [13]. It has been known as a traditional medicine that has curative properties for pain and swelling, throat and lung problems, and oral hygiene [14, 15]. PB extracts contain various bioactive phenolic compounds, such as hydroxychavicol [16], chavibetol [17], and allylpyrocatechol [18], which are regarded as having valuable biological effects. In the past decade, resurgence of interest in medicinal plants contributed more researches on the effects of PB extracts and their bioactive compounds, including wound healing [19] and antioxidant [13] antidiabetic [20], antibacterial and antifungal [21, 22], and anti-inflammatory [23] effects. Some effects of PB leaves are even being patent filed for their anticancer and immunomodulatory properties [24, 25]. Although many studies have reported the valuable effects of PB, research is still ongoing to discover the mechanisms involved, especially its role in preventing cellular aging. Hence, the present study aimed to evaluate the molecular mechanism of PB extracts in delaying replicative senescence of HDFs by focusing on its senescence-associated genes expression modulation and further insight into the link between these genes and cellular aging.

## 2. Materials and Methods

### 2.1. Cell Culture and the Induction of Replicative Senescence

This research was approved by the Research Ethics Committee of Universiti Kebangsaan Malaysia (UKMREC) (Approval Project Code: FF-289-2011). Primary HDFs were derived from foreskins of three different male subjects aged between 9 and 12 years after circumcision. Written informed consents were obtained from subjects' parents. The samples were processed and cultured as described in Makpol et al. [6]. Serial passaging was carried out with expansion degree of 1 : 4 when the culture achieved 80 to 90% confluency until HDFs reached senescence. As in previous report [6], HDFs at passage 4 (young cell, population doublings, PD* < *12), passage 15 (presenescent cell, 30* < *PD* < *40), and passage 30 (senescent cell, PD* > *55) were used for the subsequent experiments. Each stage of cells was divided into two groups, which were nontreated group and PB extracts-treated group.

### 2.2. PB Extracts Preparation

PB leaves were obtained from Kampung Lebu, Bentong, Pahang, Malaysia. The extraction method was according to Pin and colleagues [26] with some amendments. Briefly, the leaves were dried under the sun and then made up to a 10% solution by mixing 200 g of grinded leaves with 2 liters of ultrapure water. The suspension was heated to 60°C for 2 hours by using Soxhlet extractor (Thermo Scientific, UK). After that, the extracts were filtered by filter paper and kept at −20°C for 3 days. Then, by using a freeze dryer system (Labconco, USA), the PB extracts were dried into powder form and stored at 4°C. For the subsequent experiment, stock of PB extracts was prepared in complete culture medium at a concentration of 1 mg/ml followed by serial dilution to get the desired PB extracts concentration.

### 2.3. Cell Proliferation Assay

Cell proliferation assay was performed by using CellTiter 96® AQ_ueous_ Nonradioactive Cell Proliferation Assay (Promega, USA), according to the protocol given by manufacturer. This colorimetric assay was made up by 3-(4,5-dimethylthiazol-2-yl)-5-(3-carboxymethoxyphenyl)-2-(4-sulfophenyl)-2H-tetrazolium (MTS) and phenazine methosulfate (PMS). Briefly, 2 × 10^4^ of cells were plated in 96-well plate (Becton Dickinson, USA) and incubated overnight. A serial dilution of PB extracts at concentrations starting from 0.2 mg/ml until 0.8 mg/ml was prepared in culture medium and used to treat the cells. After 24-hour incubation, 20 *μ*l of MTS solution was added and cells were further incubated for 2 hours. The absorbance of the formazan produced was measured at 490 nm by using a tunable microplate reader (VersaMax Molecular Devices, USA).

### 2.4. Primer Design

Primers for human* GAPDH, SOD1, SOD2, CAT, GPX1*,* CCS, PRDX6, FOXO3*,* CDKN2A, PAK2, TP53, MAPK14*, and* JUN* were designed by using GenBank database sequences and Primer 3 software [27] (http://bioinfo.ut.ee/primer3-0.4.0/). With Basic Local Alignment Search Tool (BLAST), the primers were aligned. The efficiency and specificity of these primers were confirmed by evaluating the melt curve produced in qRT-PCR. All of the primer sequences were shown in Table 1.

### 2.5. Total RNA Extraction

TRI Reagent® (Molecular Research Center, USA) was used to extract the RNA according to manufacturer's instructions. Firstly, 1 ml of TRI reagent was added and collected into a tube after 5 minutes. To separate the cell lysate, chloroform was added and homogenized. The clear layer of the solution was collected after being centrifuged at 4°C. To precipitate the RNA, Polyacryl Carrier (Molecular Research Center, USA) was added following the isopropanol. The extracted RNA (white pellet) was washed with 75% ethanol and air-dried for 15 minutes. Sufficient amount of DNase RNase free distilled water (Gibco, USA) was added to dissolve the RNA and then stored at −80°C. By using NanoDrop ND-1000 (Thermo Scientific, USA), the yield and purity of the extracted RNA were examined.

### 2.6. Quantitative Real-Time Polymerase Chain Reaction (qRT-PCR)

The expression levels of* SOD1, SOD2, CAT, GPX1*,* CCS, PRDX6, FOXO3*,* CDKN2A, PAK2, TP53, MAPK14*, and* JUN *were quantitatively analyzed by using one-step qRT-PCR technique. Each target gene expression was normalized by reference gene,* GAPDH* [28]. The reaction was performed using 100 ng of total RNA at a concentration of 400 nM for each primer and iScript One-Step RT-PCR kit with SYBR Green (Bio-Rad, Canada) according to the manufacturer's instructions. The master mix was prepared; then reactions were carried out by using iQ5 Bio-Rad iCycler with programmed reaction profile as follows: cDNA synthesis for 30 min at 50°C; predenaturation for 2 min at 94°C; and PCR amplification for 38 cycles of 30 sec at 94°C and 30 sec at 60°C. After the end of the last cycle, the melt curve was generated at 95°C for 1 min, 55°C for 1 min, and 60°C for 10 sec (70 cycles, increase in set point temperature after cycle 2 by 0.5°C). The relative expression values of target genes were calculated using the 2^−ΔΔCt^ method.

### 2.7. Statistical Analysis

Each experiment was performed in triplicate using HDFs from three different biological subjects. Data was analyzed by one-way analysis of variance (ANOVA) followed by post hoc multiple comparison tests. A *p* value less than 0.05 (*p* < 0.05) was considered as statistically significant.

## 3. Results 

### 3.1. Dose Response Curve of PB Extracts on HDFs' Cell Proliferation

**Figure 1:**
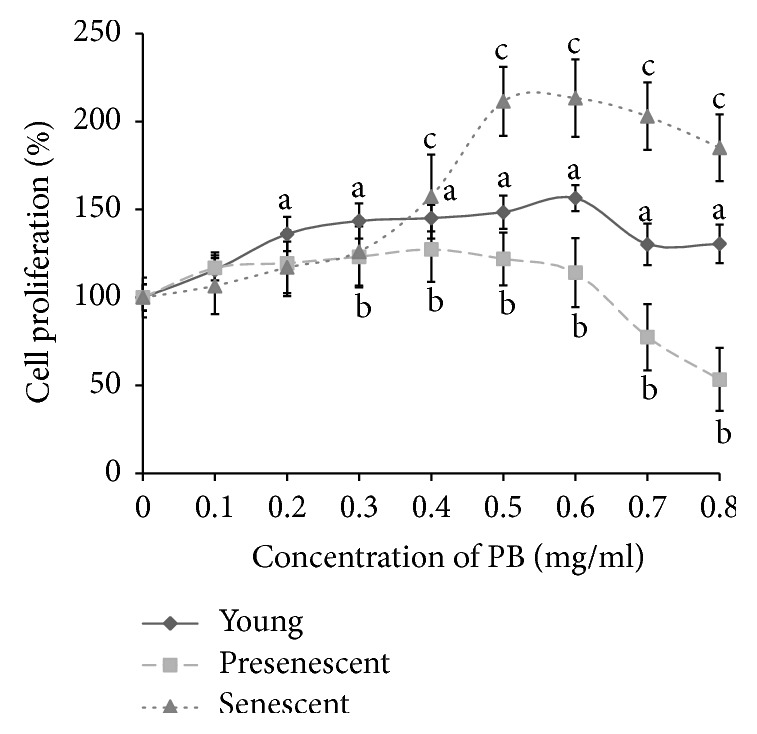
Dose response of PB extracts on proliferation of young, presenescent, and senescent HDFs. a denotes *p* < 0.05 compared to control young HDFs, b denotes *p* < 0.05 compared to control presenescent HDFs, and c denotes *p* < 0.05 compared to control senescent HDFs. Data are presented as mean ± SD (*n* = 3).

The results showed that PB extracts significantly increased the cell proliferation of young HDFs compared to control (*p* < 0.05) at concentration ranging from 0.2 mg/ml to 0.8 mg/ml (Figure 1). Meanwhile, the cell proliferation was increased in presenescent cells when treated with PB extracts at 0.3 mg/ml until 0.6 mg/ml but cell proliferation decreased at 0.7 mg/ml to 0.8 mg/ml. Senescent HDFs increased their cell proliferation when treated with PB extracts at 0.4 mg/ml until 0.8 mg/ml. Therefore, we used 0.4 mg/ml of PB extracts for the following experiments, because, at this dosage, PB increased the cell proliferation of young (143%), presenescent (127.3%), and senescent (157.3%) HDFs compared to untreated cells.

### 3.2. Effects of Replicative Senescence on Senescence-Associated Genes Expression

**Figure 2:**
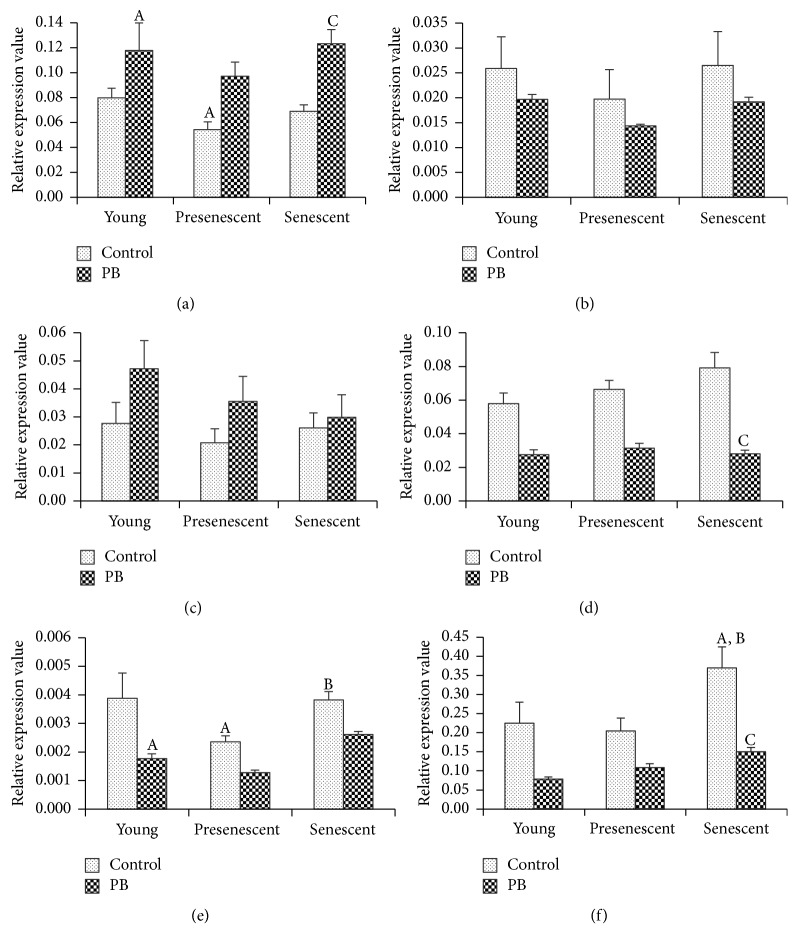
Effects of 0.4 mg/ml PB extracts on antioxidant-associated genes expression of HDFs treated for 24 hours. (a)* SOD1*, (b)* SOD2*, (c)* CAT*, (d)* GPX1*, (e)* CCS*, and (f)* PRDX6*. A denotes *p* < 0.05 compared to control for young HDFs, B denotes *p* < 0.05 compared to control for presenescent HDFs, and C denotes *p* < 0.05 compared to control for senescent HDFs. Data are presented as mean ± SEM (*n* = 3).

We determined several antioxidant-associated genes expression (*SOD1, SOD2, CAT, GPX1*,* CCS*, and* PRDX6*) in different PD stages of HDFs (Figure 2). The expression of* SOD1* in presenescent HDFs was lower as compared to young control (Figure 2(a)). However,* SOD2*,* CAT*, and* GPX1 *expressions were not significantly different between young, presenescent, and senescent groups (Figures 2(b), 2(c), and 2(d)).* CCS* was downregulated in presenescent HDFs compared to young control and significantly increased in senescent HDFs as compared to presenescent HDFs (Figure 2(e)).* PRDX6* expression was increased in senescent cells compared to both young and presenescent HDFs (Figure 2(f)).

**Figure 3:**
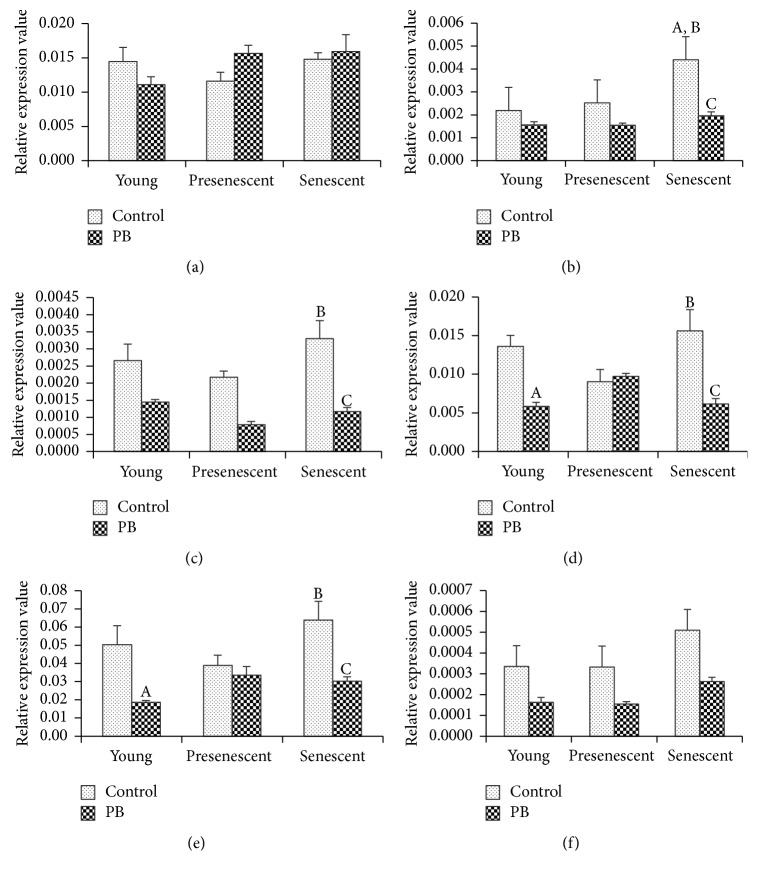
Effects of 0.4 mg/ml PB extracts on stress response genes expression of HDFs treated for 24 hours. (a)* FOXO3*, (b)* TP53*, (c)* CDKN2A*, (d)* PAK2*, (e)* MAPK14*, and (f)* JUN*. A denotes *p* < 0.05 compared to control for young HDFs, B denotes *p* < 0.05 compared to control for presenescent HDFs, and C denotes *p* < 0.05 compared to control for senescent HDFs. Data are presented as mean ± SEM (*n* = 3).

Apart from the antioxidant-associated genes expression, we also investigated several stress response genes, including* FOXO3*,* TP53, CDKN2A*,* PAK2*,* MAPK14*, and* JUN *(Figure 3). The expressions of* FOXO3 *and* JUN *were not significantly different among the 3 groups (Figures 3(a) and 3(f)). However,* TP53 *expression was significantly increased in senescent HDFs compared to young and presenescent HDFs (Figure 3(b)). Expressions of* CDKN2A, PAKK2*, and* MAPK14 *were higher in senescent HDFs as compared to presenescent cells (Figures 3(c), 3(d), and 3(e)).

### 3.3. Effects of PB Extracts on Senescence-Associated Genes Expression

Our data showed that PB extracts increased* SOD1 *expression in both young and senescent cells compared to untreated controls, respectively (Figure 2(a)). PB extracts downregulated both* GPX1 *and* PRDX6* in senescent cells compared to untreated senescent cells (Figures 2(d) and 2(f)). PB extracts treatment decreased the expression of* CCS *in young HDFs compared to untreated young cells (Figure 2(e)). Expressions of* SOD2 *and* CAT *were not different between PB extracts-treated and untreated groups (Figures 2(b) and 2(c)).

For the stress response genes, our results showed no significant change on* FOXO3* and* JUN* expressions with PB extracts treatment compared to untreated cells (Figures 3(a) and 3(f)). However, PB extracts decreased the expressions of* TP53* and* CDKN2A* in senescent HDFs compared to untreated senescent cells (Figures 3(b) and 3(c)). Treatment with PB extracts reduced* PAK2 *and* MAPK14 *expressions in both young and senescent cells compared to untreated controls, respectively (Figures 3(d) and 3(e)).

## 4. Discussion 

Aging is normally associated with oxidative stress, which then induces cellular response cascades that can be represented by transcriptional profile. Our study had focused on the effects of PB extracts on replicative senescence-associated genes expression of human diploid fibroblasts. PB extracts exert potent antioxidant properties that are able to scavenge free radicals [13, 29]. Allylpyrocatechol, the most potent phenolic compound in PB extracts, might play a role in eliminating the free radicals insults along with increased cellular antioxidants [30]. The senescence biomarker, senescence-associated *β*-galactosidase, was significantly reduced with PB extracts treatment in senescent HDFs, indicating the potential of PB extracts in regulating the process of replicative senescence [31]. The free radical scavenging activity of PB may be responsible for the revival of HDFs, especially in the oxidative damaged senescent cells. However, the molecular defense mechanism of PB extracts in senescent cells is poorly understood.

Generally, organism produces free radicals when undergoing normal oxidative metabolism. Excessive production of free radicals is expected to result in adverse changes that accumulate with age, concomitantly induceing stress signaling response in the cell. In order to compensate the oxidative damage, organism possesses an antioxidant defense mechanism that mainly is comprised of antioxidant enzymes. For instance, SODs (Cu/ZnSOD and MnSOD) in our body play a key role in eliminating superoxide radicals (O_2_^−^), while CAT and GPX1 catalyze the decomposition of hydrogen peroxide (H_2_O_2_) to water (H_2_O) and oxygen (O_2_) [9]. However, this study showed no significant change in the expressions of* SOD1 (Cu/ZnSOD), SOD2 (MnSOD), CAT*, and* GPX1* in between young and senescent cells. These findings are comparable to the findings obtained by Hazane and others [32], who observed no significant difference in antioxidant-associated genes expression in primary HDFs from three PD groups. Copper chaperone for superoxide dismutase 1 (CCS1) is required for the maintenance of redox balance by facilitating copper insertion into SOD1 [33, 34]. In this study, the role of* CCS1 *remained difficult to interpret as its expression was increased in young and senescent cells as compared to presenescent cells. PRDX6 is an alternative peroxidase that uses GSH as an electron donor to reduce H_2_O_2_ [35]. Our result showed that* PRDX6 *expression was increased in senescent HDFs, suggesting that increased* PRDX6 *expression may be needed to protect the senescent cells from oxidative stress.

The family of forkhead class O (FOXO) proteins regulate diverse physiological processes, including oxidative stress, cell cycle arrest, and apoptosis, which are mediated through a distinct forkhead DNA-binding domain [36, 37]. Downregulation of* FOXO3A* gene and protein expression by siRNA [38] and inhibition of FOXO3A by overexpression of Akt [39] have been shown to accelerate senescence in HDFs. In addition, knockout of FOXO3A promoted replicative senescence in mouse embryonic fibroblast [40] and inactive form of FOXO3A was increased in replicative senescent rat cardiac microvascular endothelial cells [41]. In this study, however,* FOXO3A* gene expression remained unchanged among 3 PD groups, suggesting that the role of* FOXO3A* during replicative senescence might depend on types of cellular models studied.

Cell growth arrest in senescent cells could be triggered by DNA damage via p53 and/or p16 pathways. In p53 pathway, DNA double-strand breaks activated p38 MAPK (encoded by* MAPK14*) which acts as a sensor for DNA damage [42]. p38 MAPK phosphorylates p53 which causes the dissociation of p53 form Mdm2 and thus promotes p53 stabilization and accumulation. Active p53 induces the expressions of* CDKN1A* and* PAK* which suppress cell cycle progression. In addition, expressions of p53 and p21 are negatively regulated by c-JUN [43]. When* CDKN2A *(encoded for p16 protein) expression is induced, p16 prevents retinoblastoma (pRB) phosphorylation and activates it to bind with E2F transcription factor and thus promotes cell cycle arrest [44]. Previously, we have shown that DNA damage was accumulated and cell growth was arrested at G1 phase during replicative senescence of HDFs [6]. Our results in this study showed that expressions of* MAPK14*,* TP53*,* PAK2*, and* CDKN2A* were increased in senescent cells, suggesting that p53 and p16 pathways may be involved in the cell growth arrest of replicative senescent HDFs.

This study showed that treatment with PB extracts increased* SOD1* expression in senescent cells, which is similar to the findings on an animal study which reported that the activity of liver SOD increased after 2 weeks of oral supplementation of PB extracts [45]. In Nrf2 (transcription factor for SOD1) knockout mouse embryonic fibroblast, PB extracts were able to induce* SOD1* gene expression [46]. Our data suggested that PB extracts may be involved in cellular antioxidant defense system of replicative senescent cells by regulating gene expression of antioxidant enzyme. Conversely, PB extracts treatment reduced the expressions of* GPX1 *and* PRDX6* in senescent HDFs. In agreement with Dasgupta and De [13], PB extracts have free radical scavenging property, which reduces lipid peroxidation. Thus, the presence of this potent free radical scavenger in PB-treated cells may lead to the decreased need on endogenous antioxidant enzymes.

PB was able to prevent radiation-induced DNA damage and promote cell proliferation [17]. Allylpyrocatechol and chavibetol in PB extracts were reported to be able to protect liver mitochondria from photosensitization-induced lipid peroxidation; meanwhile, allylpyrocatechol alone was found to inhibit type II photosensitization damage in fibroblasts [18]. This proposed that the active compounds in PB extracts could eliminate the stress stimulants in cells. Our data showed that* TP53, CDKN2A, PAK2*, and* MAPK14 *expressions were decreased in senescent cells treated with PB extracts, indicating that DNA damage via p53 and p16 pathways is suppressed. These data suggest that DNA damage and cell cycle arrest may be ameliorated in senescent cells due to the reduced oxidative stress.

**Figure 4:**
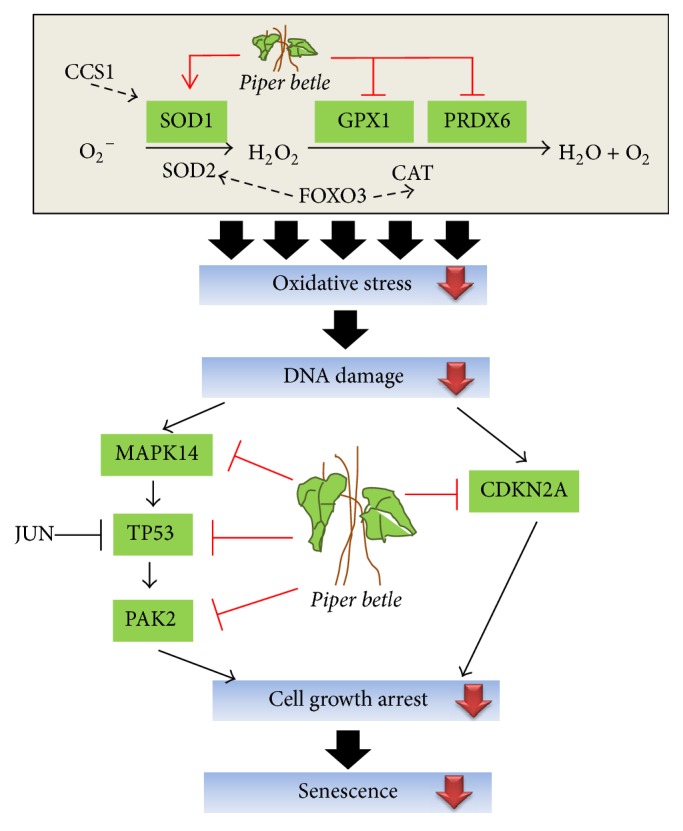
An illustration on the mechanism of PB extracts in ameliorating replicative senescence of HDFs. PB extracts upregulate* SOD1* expression which results in reduced oxidative stress. Reduced oxidative stress spares the expressions of* GPX1 *and* PRDX6*, decreases DNA damage, suppresses gene expressions of* TP53* and* CDKN2A* (p16) signaling pathways, promotes cell cycle progression, and thus increases cell proliferation of senescent HDFs.

Taken together, as illustrated in Figure 4, we proposed that PB extracts increased the proliferation of senescent cells by alleviating the oxidative stress during replicative senescence via increasing of* SOD1 *expression to scavenge free radicals. Treatment with PB extracts reduced the expressions of* GPX1 *and* PRDX6*, suggesting that the needs on endogenous antioxidant enzymes may be reduced. After enhancing the antioxidant status, the extent of DNA damage and cell cycle arrest will be decreased and thus results in the downregulation of* TP53, CDKN2A*,* PAK2*, and* MAPK14 *expressions.

## 5. Conclusion

PB extracts modulate the expressions of gene involved in antioxidant defense (*SOD1*,* GPX1*, and* PRDX6*), DNA damage, and cell cycle arrest (*TP53*,* CDKN2A, PAK2*, and* MAPK14*) signaling pathways during replicative senescence of HDFs. Further studies are needed to characterize the active compounds in PB extracts which are responsible for the gene regulation during the replicative senescence of HDFs and to determine the functional roles of these genes in mediating the effect of PB during replicative senescence of HDFs.

## Figures and Tables

**Table 1:** Primer sequences for quantitative real-time PCR.

Target genes	Forward primer (5′-3′)	Reverse primer (5′-3′)	Product size (bp)
*GAPDH *	tcc ctg agc tga acg gga ag	gga gga gtg ggt gtc gct gt	217
*SOD1 *	gaa ggt gtg ggg aag cat ta	aca ttg ccc aag tct cca ac	174
*SOD2 *	cgt cac cga gga gaa gta cc	ctg att tgg aca agc agc aa	312
*CAT *	cgt gct gaa tga gga aca ga	agt cag ggt gga cct cag tg	119
*GPX1 *	cca agc tca tca cct ggt ct	tcg atg tca atg gtc tgg aa	127
*PRDX6*	cgt gtg gtg ttt gtt ttt gg	tgc tgt cag ctg gag aga ga	120
*CCS *	act tta acc ctg atg gag cat ct	agg tca tct tct ccc tca tca at	181
*FOXO3*	gca agc aca gag ttg gat ga	cag gtc gtc cat gag gtt tt	185
*TP53 *	gga aga gaa tct ccg caa gaa	agc tct cgg aac atc tcg aag	177
*PAK2*	gat ggc acc aga ggt ggt ta	tcc cga aat att ggg gaa ag	198
*CDKN2A*	agt gag ggt ttt cgt ggt tca c	cca tca tca tga cct ggt ctt cta	150
*MAPK14*	ggg gca gat ctg aac aac at	gag cca gtc caa aat cca ga	190
*JUN *	gtc tac gca aac ctc agc aac	act gtc tga ggc tcc tcc ttc	191

## Abbreviations

*AP-1*: Activator protein 1
CDK: Cyclin-dependent kinase
*FOXO3*: Forkhead box O 3
*GADPH*: Glyceraldehyde 3-phosphate dehydrogenase
GSH: Reduced glutathione
GSSG: Oxidized glutathione
HDFs: Human diploid fibroblasts
MAPK: Mitogen-activated protein kinase
PAK: p21-activated kinase
PD: Population doubling
*PRDX6*: Peroxiredoxin 6
qRT-PCR: Quantitative real-time polymerase chain reaction
SIPS: Stress-induced premature senescence.
